# Genome-wide association study of corneal biomechanical properties identifies over 200 loci providing insight into the genetic etiology of ocular diseases

**DOI:** 10.1093/hmg/ddaa155

**Published:** 2020-07-27

**Authors:** Mark J Simcoe, Anthony P Khawaja, Pirro G Hysi, Christopher J Hammond, UK Biobank Eye and Vision Consortium

**Affiliations:** 1 Department of Ophthalmology, Kings College London, London, UK, SE1 7EH; 2 Department of Twins Research and Genetic Epidemiology, Kings College London, London, UK, SE1 7EH; 3 NIHR Biomedical Research Centre, Moorfields Eye Hospital NHS Foundation Trust and UCL Institute of Ophthalmology, London, UK, EC1V 2PD; 4 Department of Public Health and Primary Care, Institute of Public Health, University of Cambridge School of Clinical Medicine, Cambridge, UK, CB1 8RN; 5 Institute of Ophthalmology, University College London, London, United Kingdom, EC1V 9EL; 6 Institute of Child Health, University College London, London, United Kingdom, WC1N 1EH

## Abstract

Corneal hysteresis and corneal resistance factor are parameters that reflect the dynamic biomechanical properties of the cornea and have been shown to be biomarkers of corneal disease. In this genome-wide association study of over 100 000 participants, we identified over 200 genetic loci, all but eight novel, significantly associated with either one or both of these traits. In addition to providing key insights into the genetic architecture underlying normal corneal function, these results identify many candidate loci in the study of corneal diseases that lead to severe visual impairment. Additionally, using Mendelian randomization, we were able to identify causal relationships between corneal biomechanics and intraocular pressure measurements, which help elucidate the relationship between corneal properties and glaucoma.

## Introduction

Corneal hysteresis (CH) and corneal resistance factor (CRF) are quantitative measures of corneal biomechanics. Both phenotypes are measured using the ocular response analyzer (ORA), a non-contact tonometer (1) that differs from conventional non-contact tonometry as it records both the inward and outward applanation pressures, from which CH and CRF measures are derived (see Materials and Methods). CH is reflective of the viscoelastic dampening properties of the cornea (1) and is also considered indicative of corneal viscosity (2). CRF is designed to reflect the cornea’s resistance to deformation (3); it is adjusted to be maximally associated with central corneal thickness (CCT) and minimally associated with intraocular pressure (IOP). It has been proposed that corneas with reduced dampening capabilities are more susceptible to several ocular diseases (2).

CH and CRF have been implicated in several conditions of the eye. Previous work (4–6) has suggested that CH and CRF may be predictors of glaucoma, independent of elevated IOP. CH and CRF are also correlated, both phenotypically and genotypically with CCT and keratoconus (7). CH and CRF are also lower in patients with Fuchs endothelial corneal dystrophy (FECD) (8).

Genetic effects play a large role in CH and CRF variation. CH is highly heritable, with a reported narrow sense heritability of *h*^2^ = 0.77 (9). Additionally, several loci associated with both CH and CRF have been identified in a previous study (7).

This study aimed to explore the genetic architecture of CH and CRF and clarify their genetic correlation with eye and systemic disease. Participants from the UK Biobank were used as a discovery sample in this study. The UK Biobank is a population-based cohort with 503 325 participants between 40 and 69 years of age (10).

## Results

There were 106 041 and 106 030 participants with a 97.7% sample overlap (103 591 participants) included for CH and CRF analyses, respectively, following quality control (see Materials and Methods). The mean age of participants was 57.3 (SD = 7.87), and 53.2% of participants were female. A summary of the participants measured phenotypes, stratified by age group, is provided in Table 1 and the phenotypic distributions stratified by sex are displayed in Supplementary Material, Figure S1a and b. CH distributions and phenotypic associations within the UK Biobank have also been previously described elsewhere (11).

**Table 1:** Summary of phenotypic measurements, stratified by age group, of participants in the UK Biobank cohort included in these analyses

Age group	CH			CRF		
	Mean (SD)/mmHg	% female	*N*	Mean (SD)/mmHg	% female	*N*
40–49	11.01 (1.70)	54.6	21 063	10.88 (1.89)	54.6	21 060
50–59	10.78 (1.68)	55.6	34 150	10.80 (1.84)	55.6	34 146
60–70	10.43 (1.66)	50.9	50 828	10.63 (1.80)	50.9	50 824
All	10.68 (1.69)	53.2	106 041	10.73 (1.84)	53.2	106 030

**Figure 1:**
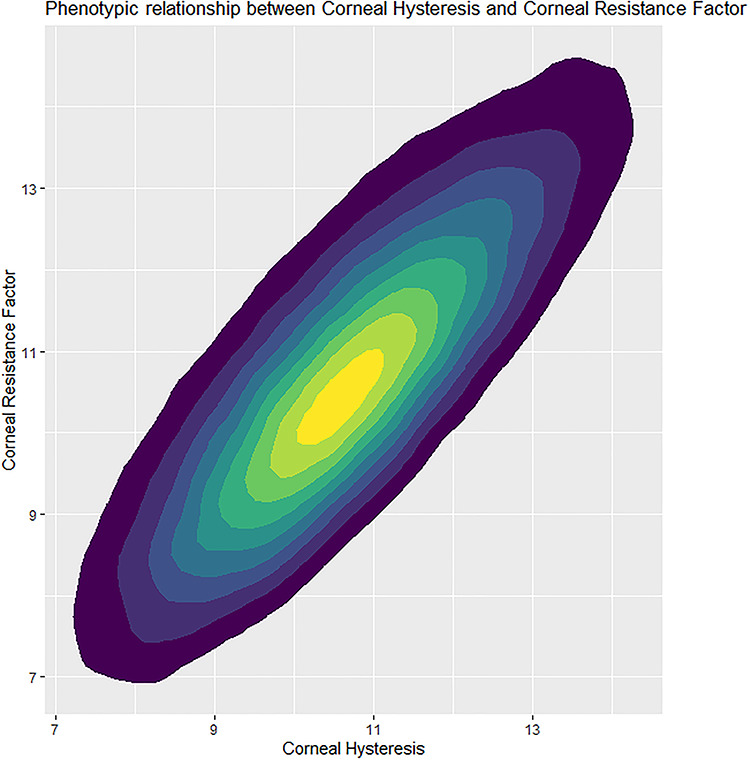
A heatmap of CH against CRF measurements for the 103 591 participants that had measurements for both post-QC.

Phenotypically, CH and CRF were highly correlated (*r* = 0.82, *P* < 1.0 × 10^−100^) (Fig. 1). Both CH and CRF are associated with participant age: CH—β = −0.031 mmHg/year (95% CI = [−0.030, −0.032], *P* < 1.0 × 10^−100^), CRF—β = −0.015 mmHg/year (95% CI = [−0.014, −0.016], *P* = 1.6 × 10^−93^), and on average men had lower CH (β = −0.40 mmHg, 95% CI = [−0.38, −0.42], *P* < 1.0 × 10^−100^) and CRF (β = −0.30 mmHg, 95% CI = [−0.28, −0.32], *P* < 1.0 × 10^−100^) than women.

CH was negatively correlated with corneal-compensated intraocular pressure (IOPcc) (*r* = −0.46, *P* < 1.0 × 10^−100^). CRF was positively correlated with IOPcc, albeit only to a small degree (*r* = 0.081, *P* < 1.0 × 10^−100^), despite CRF being adjusted to remove correlation with IOPcc (3).

Both CH and CRF were lower in primary open-angle glaucoma (POAG) cases than controls (Welch two-sample *t*-test: CH—cases *x-* = 9.96 mmHg, controls *x-* = 10.68 mmHg, *P* = 1.7 × 10^−66^; CRF—cases *x-* = 10.72 mmHg, controls *x-* = 10.92 mmHg, *P* = 1.5 × 10^−5^), despite 60.2% of POAG cases taking IOP-lowering medication, which has been reported to increase CH (12).

**Figure 2:**
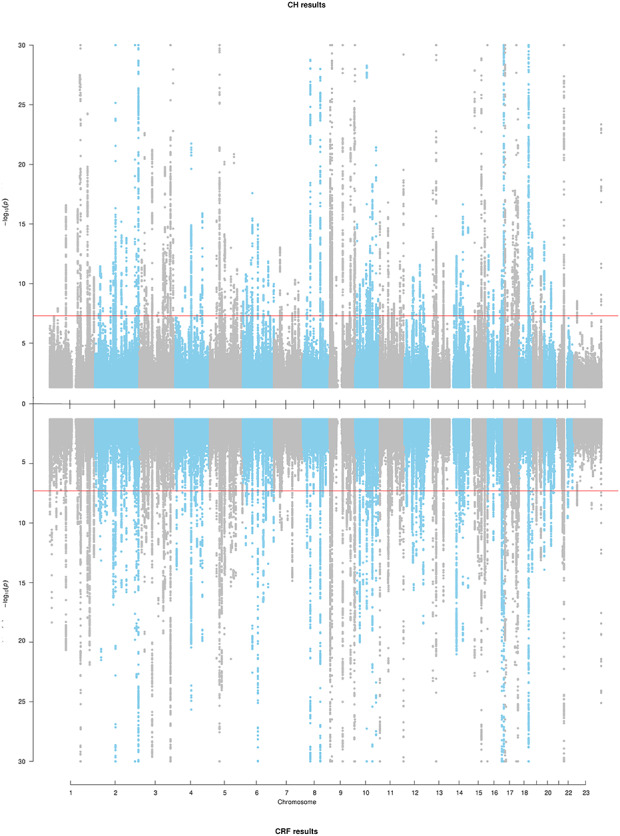
A Miami plot for CH (top) and CRF (bottom). Red line is set at genome-wide significance (*P* = 5 × 10^−8^). Minimum *P*-value was set at *P* = 1 × 10^−30^ for better graphical representation.

**Figure 3:**
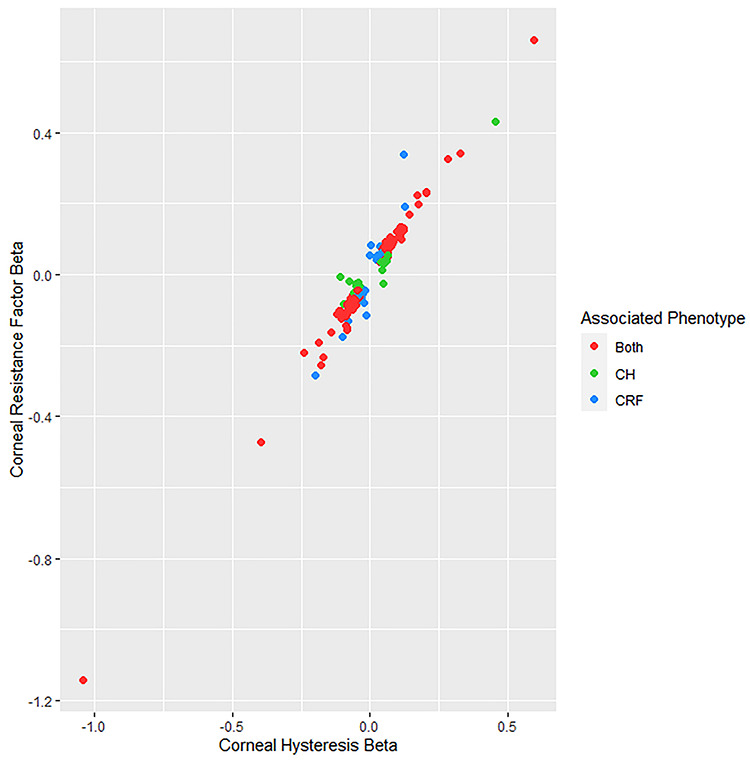
A plot of the effect sizes for the lead SNPs from each region associated with either CH or CRF. SNPs are color-coded to indicate which trait they are associated with at genome-wide significance (*P* < 5 × 10^−8^).

Over 12 million SNPs (see Materials and Methods) were tested for association separately for CH and CRF. The genomic inflation factors (13) were λ_CH_ = 1.31 and λ_CRF_ = 1.43, and the respective LD score regression intercepts (and ratios) were 1.06 (0.10) and 1.08 (0.11), consistent with expectations of polygenicity and large sample sizes (14) indicating thet results are unlikely to be inflated due to population structure. Genome-wide significant association (*P* < 5 × 10^−08^) with CH and CRF was observed for 13 945 and 20 020 variants, respectively (Miami plot of results in Fig. 2), clustered within 157 and 181 distinct genomic regions, respectively (Supplementary Material, Tables S1 and S2), including eight regions previously reported for CH and CRF (7); three of these loci (*ZNF469, TCF4* and *COL6A1*) were previously associated only with CRF but were associated with both phenotypes in our analyses. Overall, in these analyses, 111 regions were associated at genome-wide significance with both CH and CRF. Figure 3 plots the effect size of the lead SNP for CH and CRF from each genomic region, and whether they are significantly associated with either one or both CH and CRF.

Strong association was detected for variants within or adjacent to multiple genes from the collagens superfamily; association was detected for both phenotypes at loci located within the genes encoding all three subunits of collagen type VI (*COL6A1*, rs142493024, *p*_CH_ = 7.5 × 10^−98^, *p*_CRF_ = 1.1 × 10^−101^; *COL6A2*, rs1042917, *p*_CH_ = 9.7 × 10^−28^, *p*_CRF_ = 1.3 × 10^−38^; *COL6A3*, rs2645773, *p*_CH_ = 6.6 × 10^−31^, *p*_CRF_ = 4.5 × 10^−36^). Collagen type VI accounts for up to a quarter of the cornea’s dry weight (15).

Collagen type VIII is an important component of the Descemet membrane (16) in the posterior section of the cornea and has two subunits encoded by the genes *COL8A1* and *COL8A2*. SNPs upstream of *COL8A1* (rs2256606, *P* = 2.2 × 10^−9^) and within *COL8A2* (rs4653159, *P* = 2.9 × 10^−11^) were associated with CRF. However, association of these markers did not reach genome-wide significance for CH (*P* = 5.1 × 10^−7^ and 5.8 × 10^−4^, respectively).

The stroma constitutes 90% of the corneal thickness and is primarily a complex of type I and type V collagen (17). Significant association was identified near one of type I collagen’s two subunits (*COL1A1*) for CRF (rs180976308, *P* = 2.0 × 10^−11^), though for CH, this marker only had suggestive association (*P* = 2.5 × 10^−6^). Meanwhile, SNPs adjacent to the gene for one of type V collagen’s subunits were associated with both CH and CRF (rs3132302, *p*_CH_ = 9.0 × 10^−17^, *p*_CRF_ = 9.4 × 10^−28^).

Interestingly, a number of loci were specifically associated with CH and not CRF. These specific loci included several loci previously reported to be associated with IOP (18) such as: *TMCO1* (rs7518099, *p*_CH_ = 3.2 × 10^−28^, *p*_CRF_ = 0.36), *CAV2* (rs10233003, *p*_CH_ = 5.7 × 10^−11^, *p*_CRF_ = 0.001), and *DGKG* (rs9853115, *p*_CH_ = 1.1 × 10^−28^, *p*_CRF_ = 0.01). These phenotype-specific loci likely result from differences in phenotypic correlation between IOP with CH and CRF. Meanwhile, the CRF-specific loci are primarily collagen-related genes (as previously discussed), likely as a result of variation in the cornea’s physical structure having a greater influence over CRF than CH.

Additionally, loci connected with rare Mendelian disorders were associated with these corneal phenotypes, for example, a locus within the gene *VCAN* (rs149879035, *p*_CH_ = 1.3 × 10^−9^, *p*_CRF_ = 7.4 × 10^−9^). Mutations within *VCAN* are responsible for the autosomal dominant vitreoretinopathy Wagner syndrome (19). A key feature of Wagner syndrome is an abnormal structure in the vitreous gel, reducing its viscosity. Though CH is indicative of corneal viscosity, it is likely that the viscosity in both the anterior and posterior chambers has some shared genetic pathways.

Several other genes harboring associated SNPs are connected to rare genetic disorders, particularly corneal, ocular, and syndromic disorders of connective tissue (e.g. brittle cornea syndrome and Shprintzen–Goldberg syndrome) based on data available from OMIM (20) (summarized in Supplementary Material, Tables S3 and S4).

Finally, many loci known to be associated with corneal diseases, including keratoconus (21) and FECD (22), were strongly associated with CH and CRF. These included known keratoconus loci: *FNDC3B* (rs7635832, *p*_CH_ = 6.7 × 10^−91^, *p*_CRF_ = 6.9 × 10^−134^), *ZNF469* (rs28425635, *p*_CH_ = 4.3 × 10^−92^, *p*_CRF_ = 1.5 × 10^−109^)*, MPDZ* (rs12686184, *p*_CH_ = 7.0 × 10^−86^, *p*_CRF_ = 5.6 × 10^−96^) and *FOXO1* (rs11616662, *p*_CH_ = 1.7 × 10^−74^, *p*_CRF_ = 3.5 × 10^−84^); and FECD loci: *TCF4* (rs11659764, *p*_CH_ = 2 × 10^−77^, *p*_CRF_ = 3.9 × 10^−92^), *ATP1B1* (rs1200108, *p*_CH_ = 6.2 × 10^−53^, *p*_CRF_ = 2.8 × 10^−41^) and *SLC25A22* (rs12223324, *p*_CH_ = 2.4 × 10^−15^, *p*_CRF_ = 1.1 × 10^−31^).

Conditional analysis refined these results, identifying 203 and 258 informative SNPs associated with CH and CRF, respectively. The Ensembl Variant Effect Predictor (23) identified that out of the 203 conditional SNPs for CH, 12 are missense variants and 39 are regulatory region variants (Supplementary Material, Table S5). Of the 258 conditional SNPs for CRF, 15 are missense and 40 are in regulatory regions (Supplementary Material, Table S6). Seven missense variants: rs72755233 (*ADAMTS17*), rs12448432 (*HAGHL*), rs77542162 (*ABCA6*), rs77583146 (*WNT10A*), rs121908120 (*WNT10A*), rs1042917 (*COL6A2*) and rs139498917 (*MAMDC2*) were independently associated with both phenotypes; their missense coding effects make them strong candidates for causal loci. The minor allele for one of these SNPs in particular, rs139498917_A (*MAMDC2*), is associated with a very large, and clinically relevant increase in both CH and CRF (β_CH_ = 1.04, SE = 0.07; β_CRF_ = 1.14, SE = 0.08), equal to a 0.62 standard deviation increase per allele for both.

**Figure 4:**
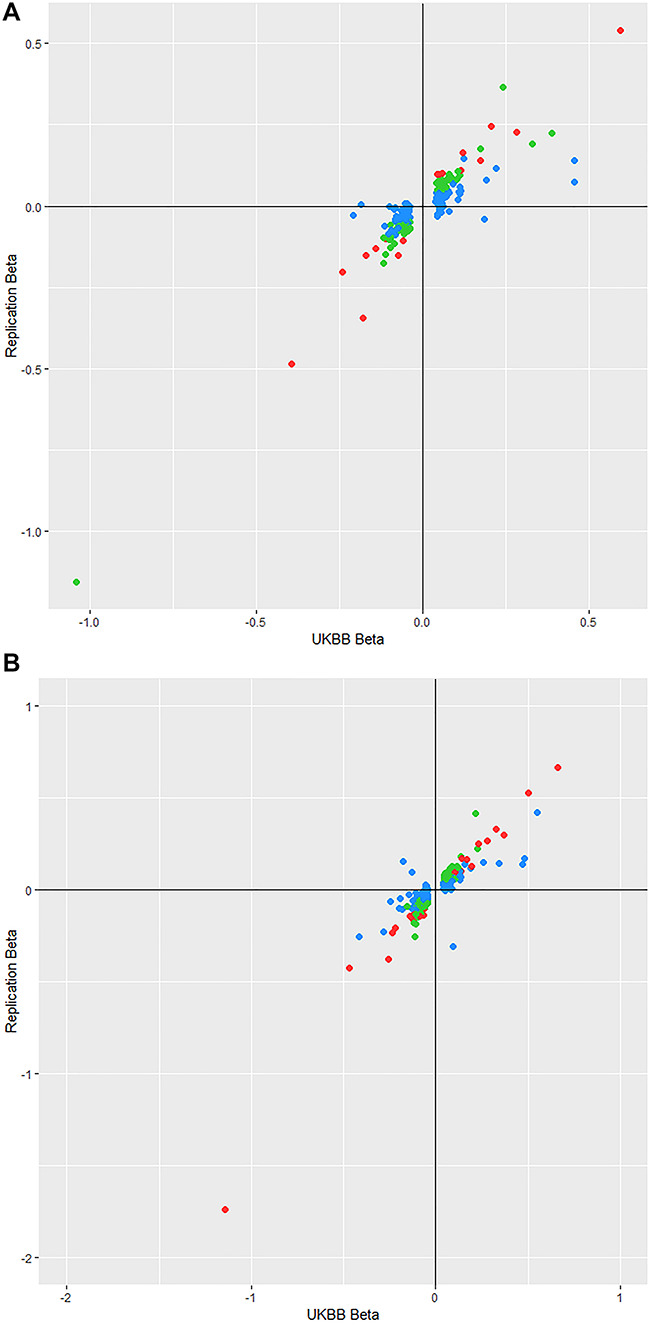
(**A**) Plot of conditional SNP effect sizes for CH between the discovery and replication cohorts. Points are color-coded by their *P*-value in the replication cohort where red points reach Bonferroni-corrected significance (*P* < 2.5 × 10^−4^), green points have nominal significance (*P* < 0.05) and blue have *P* > 0.05. (**B**) Plot of conditional SNP effect sizes for CRF between the discovery and replication cohorts. Points are color-coded by their *P*-value in the replication cohort where red points reach Bonferroni-corrected significance (*P* < 1.98 × 10^−4^), green points have nominal significance (*P* < 0.05) and blue have *P* > 0.05.

Replication of the autosomal conditional SNPs (200 SNPs for CH and 253 for CRF) was conducted with a meta-analysis of two independent cohorts: TwinsUK and EPIC-Norfolk, as previously described (7). Though the replication dataset was less than 10% of the discovery cohort sample size, 17 SNPs from 17 separate regions replicated at Bonferroni-adjusted significance (*P* < 2.5 × 10^−4^) (Supplementary Material, Table S7) for CH, while an additional 94 SNPs were associated with nominal significance (*P* < 0.05) with the same direction of effect. Two SNPs (rs534975221 and rs544072714) were not present in the replication dataset for CRF. From the remaining 253 markers that were tested, 26 SNPs from 20 distinct regions replicated at Bonferroni-adjusted significance (*P* < 1.98 × 10^−4^) (Supplementary Material, Table S8), while an additional 106 SNPs had an equal direction of effect with at least nominally significant association. There was very strong correlation of effect sizes between the discovery and replication datasets: *r*_CH_ = 0.90, *P* = 3.6 × 10^−73^; *r*_CRF_ = 0.89, *P* = 2.5 × 10^−86^. Figure 4a and b show plots of the effect sizes between datasets for CH and CRF, respectively.

In line with our observations of association for polymorphisms within or near several collagen-coding genes, gene-set enrichment analyses (after adjustment for 0.05 false discovery rate (FDR)) identified enrichment for collagen pathways including the ‘Collagens’ (24) gene set (FDR adjusted *q*_CH_ = 9.0 × 10^−4^, *q*_CRF_ = 9.1 × 10^−3^). There was noticeable enrichment for gene sets of systemic pathways such as the Gene Ontology (25) ‘Skeletal System Development’ (FDR-adjusted *q*_CH_ = 6.0 × 10^−4^, *q*_CRF_ = 0) (all results are summarized in Supplementary Material, Tables S9 and S10).

Currently, eQTL data for corneal tissue are not publicly available; however, within the cornea, there is a high density of keratocytes (26), a sub-type of fibroblast. Therefore, eQTL data for fibroblasts from the GTEx database (27) were used as a surrogate reference for eQTL effects within the cornea. To check the validity of eQTL-derived results, RNA-seq data (28) were used to check if the implicated mRNAs are expressed in corneal tissue. Significant eQTL effects in fibroblasts were present for 70 CH conditional SNPs and 82 CRF SNPs; 58/70 CH eQTL mRNAs and 69/82 for CRF are known to be expressed within corneal tissue (Supplementary Material, Tables S11 and S12), the mRNAs for the other eQTLs were not included in Carnes *et al.*’s (28) RNA-seq analysis, so it is unknown whether or not they are expressed in corneal tissue.

Using the same GTEx (27) fibroblast transcriptome dataset as the previous analysis, we performed gene-level association testing with S-Predixcan (29) for both CH and CRF. The strongest gene association for both phenotypes was increased expression of *ZNF469* (*p*_CH_ = 1.7 × 10^−40^, *p*_CRF_ = 6.5 × 10^−52^) and a non-coding RNA (*RP11-863P13.3*: *p*_CH_ = 9.7 × 10^−31^, *p*_CRF_ = 3.7 × 10^−34^), upstream of *ZNF469* on chromosome 16 (Supplementary Material, Tables S13 and S14). These results indicate that the strong association in the SNP-based GWASs for variants within *ZNF469* (rs28425635, *p*_CH_ = 2.0 × 10^−92^, *p*_CRF_ = 1.5 × 10^−109^) is likely mediated through variation in gene expression. In total, there was significant association for expression of 86 genes with CH, and 107 with CRF, while 75/86 of the CH-associated genes and 96/107 of the CRF-associated genes are known to be expressed within corneal tissue (Supplementary Material, Tables S13 and S14).

Genetic correlations (denoted *r*_g_) were identified for CH and CRF with multiple phenotypic traits (Supplementary Material, Tables S15 and S16). As expected, their strongest correlation was with each other (*r*_g_ = 0.88, *P* = 2.2 × 10^−1547^) and with CCT (CH: *r*_g_ = 0.64, *P* = 8.0 × 10^−32^; CRF: *r*_g_ = 0.69, *P* = 1.3 × 10^−45^). Systemic trait correlations for CH and CRF were similar, such as forced vital capacity (CH: *r*_g_ = −0.12, *P* = 3.1 × 10^−7^; CRF: *r*_g_ = −0.10, *P* = 1.2 × 10^−5^) and standing height (CH: *r*_g_ = −0.11, *P* = 2.7 × 10^−7^; CRF: *r*_g_ = −0.09, *P* = 3.3 × 10^−5^). However, IOPcc correlations with CH and CRF were in reverse directions (CH: *r*_g_ = −0.18, *P* = 1.5 × 10^−10^; CRF: *r*_g_ = 0.34, *P* = 6.8 × 10^−42^) and for POAG (CH: *r*_g_ = −0.14, *P* = 7.0 × 10^−3^; CRF: *r*_g_ = 0.30, *P* = 1.4 × 10^−10^). The genetic correlation with IOPcc and POAG was stronger for CRF than CH, despite the reverse being true for IOPcc phenotypically.

In light of the genetic correlations between IOPcc and both CH and CRF, despite IOPcc and CRF having minimal phenotypic correlation, we applied Mendelian randomization (30) to infer any direct causal relationships. A combination of Mendelian randomization approaches indicated that the ‘penalized’ regression models were most appropriate for these data (see Materials and Methods for details). Results infer that IOPcc has a direct causal effect on both corneal biomechanical phenotypes (best-fitting models summarized in Table 2). However, when testing for causality in the opposite direction (CH and CRF affecting IOPcc), the MR-Egger intercept was significantly different from 0. These results indicate that IOPcc has a unidirectional causal effect over CH and CRF and that IOP influences how the cornea responds to biomechanical change. Full results from all models are provided in Supplementary Material, Tables S17–S20.

**Table 2:** Results from the best fitting models for Mendelian randomization

Exposure phenotype	Outcome phenotype	Number of SNPs	Model	Beta	SE	*P*-value
CH	IOPcc	144	IVW	−0.128	0.024	<1E-3
			Egger	0.048	0.065	0.462
			Egger intercept	−0.011	0.004	0.014
CRF	IOPcc	166	IVW	0.31	0.021	<1E-3
			Egger	0.005	0.054	0.928
			Egger intercept	0.018	0.004	<1E-3
IOPcc	CH	99	IVW	−0.154	0.009	<1E-3
			Egger	−0.153	0.032	<1E-3
			Egger intercept	0.002	0.004	0.537
IOPcc	CRF	96	IVW	0.137	0.01	<1E-3
			Egger	0.12	0.031	<1E-3
			Egger intercept	0.002	0.004	0.54

For the model, IVW is the penalized inverse weighted variance, Egger is the penalized Egger model and Egger intercept is the corresponding intercept results for the given Egger results shown. Number of SNPs is the number of uncorrelated SNPs used as genetic instruments for each test.

## Discussion

This GWAS identified a total of 217 distinct genomic regions significantly associated with either one or both corneal biomechanical properties, CH and CRF. These include loci implicated in the corneal diseases of keratoconus (21) and FECD (22), alongside many novel associations, and replicated all previously reported associations for these phenotypes (7).

One of these novel associations was on chromosome X, which is often excluded from GWAS analyses (31). The strongest association on this chromosome was within the biglycan gene (*BGN*). The biglycan protein forms a complex acting as a link between type II and VI collagen, with biglycan-deficient animal models having disarrayed collagen fibrils (32). Association at this locus is another component in collagen-related pathways, which were highly enriched in our results.

Previous studies have shown that CH and CRF are significantly different in patients with corneal dystrophies such as keratoconus (33,34) and FECD (8) compared to healthy controls, and in the case of keratoconus, they have a strong predictive value for disease status (34). However, due to these conditions having a reasonably low prevalence, it is difficult to acquire large numbers of cases to provide statistical power in GWAS analyses. Therefore, CH and CRF serve as useful endophenotypes, and the numerous significantly associated loci in our analyses provide suitable candidates for further study of the genetic etiology of corneal diseases.

There is an enrichment of associated variants within or adjacent to genes for multiple collagen subunits for both CH and CRF. As different collagen types are expressed throughout different layers of the cornea, these results indicate that variations in collagens throughout the corneal layers influence corneal biomechanical properties. Interestingly, despite analyzing the same sample for both phenotypes, there is both a greater enrichment of collagen loci and a greater number of significantly associated loci for CRF than for CH. This is possibly a consequence of IOP effects on the corneal dampening capabilities; thus, CH measurements contain an IOP component, and IOP is known to be a relatively ‘noisy’ phenotype (35). Meanwhile, CRF was designed to have a minimal IOP component (3), which in turn may reduce noise in the data.

Mendelian randomization indicates that IOPcc exerts causal effects over CH and CRF. The directions of effect show that an increase in IOPcc will reduce CH but simultaneously raise CRF. Although counterintuitive at first sight given the strong phenotypic correlation between CH and CRF (*r* = 0.82, *P* < 1.0 × 10^−100^), these results are consistent with the core properties of these parameters. CH is representative of the cornea’s dampening capabilities which will be reduced by increased pressure, whereas CRF represents the resistance within the cornea which will increase when subjected to raised pressure. Interestingly, these results infer that CRF is not independent of IOP, despite the formula to derive it being designed to minimize IOP correlation and effects (3); genetic correlation between CRF and POAG (*r*_g_ = 0.30, *P* = 1.4 × 10^−10^) is likely driven by the effect IOP has over CRF. In light of these results, further study is required to determine the utility and independence of CH as a risk factor for POAG progression (4).

This study is the largest to date, and uniform measurement methods were used in a largely homogenous UK population. Our findings provide new insight into the genetic architecture of dynamic corneal properties, which may be useful in aiding our understanding of both normal corneal development and disease pathways involved in a number of corneal conditions.

## Materials and Methods

We performed a GWAS using information from the 117 649 participants that took part in the eye and vision component in the UK Biobank (10).

### Subjects

Subjects included for this analysis provided full informed consent in accordance with ethical approval granted and overseen by the UK Biobank Ethics and Governance Council. All subjects included were confirmed to be of European ancestry through principal component analysis as described in a previous study (18), and all first-degree relatives, as determined by identity by descent calculations, were excluded.

### Phenotyping

CRF and CH were both measured using the ORA device, during an eye examination performed by a trained technician. The ORA uses a 20 ms air pulse to flatten the cornea, while using an electro-optical system to measure the inward (designated P1) and outward (designated P2) applanation pressures (1). CH is defined as the absolute difference between P1 and P2 (1), while CRF is a linear function of P1 and P2 that has been adjusted to minimize correlation with corneal-compensated intraocular pressure (IOPcc) while maximizing correlation with CCT (3). Measurements were excluded from analysis if the participants had a history of glaucoma or corneal surgery, refractive surgery, eye injuries, and those currently using glaucoma medications. In addition, the top and bottom 0.5 percentiles were excluded to remove any outliers that are likely artefact. The mean of measures from the left and right eye was used as the outcome variable. In total, 106 041 and 106 030 participants remained for CH and CRF analysis, respectively, with a 97.7% sample overlap (103 591).

POAG cases and controls were ascertained using ICD10 codes and self-reported questionnaire data. Use of IOP-lowering medication was self-reported.

### Genotyping

UK Biobank participants were genotyped on one of two arrays: the Affymetrix UK Biobank Axiom array, and the UK BiLEVE array. Imputation of additional variants was performed using a combined Haplotype Reference Consortium (HRC) (36) and UK10K (37) reference panel; full details for genotyping and imputation are described elsewhere (38). Only variants that were either directly genotyped or imputed using the HRC reference panel were included in our analysis. Variants were then filtered with minor allele frequency >0.1% and an imputation quality score >0.4 cut-off.

### Association tests

The CH and CRF measurements were used as the outcome variables in separate linear mixed model regressions, under the assumption of an additive model for allelic effects. Adjustments were made for age, sex and the first five principal components. These analyses were performed in BOLT-LMM (39) using a linear mixed model, that provides additional corrections for population structure and cryptic relatedness. For chromosome X, genotypes were coded as diploid (males coded as 0/2 and females as 0/1/2) to account for dosage compensation.

### Phenotypic association tests

Phenotypic associations with age were tested in R (40) using a linear model, adjusted for age and sex. Correlation between CH and CRF was tested with Pearson’s correlation coefficient.

### Genomic region and significance specification

In this paper, an ‘associated region’ was defined as a region with genome-wide significantly associated markers, separated from other associated regions by a minimum of 1 million base-pairs that are not significantly associated. Genome-wide significance for our study was set at *P* < 5 × 10^−8^, customary for these analyses.

### LD score regression and genetic correlation

An LD score regression intercept was calculated to identify any possible inflation (14) using the LD Hub (41). This approach is more informative for this study than the Devlin genomic inflation lambda (13), on account of the large cohort size and high trait polygenicity. LD Hub was then used to test for genetic correlation between other heritable traits, and CH and CRF, respectively (42).

### Conditional analysis

Following the same procedure outlined by Yang *et al.* (43), conditional analysis was conducted in GCTA to identify variants that are independently associated with CH or CRF.

### Gene pathway enrichment analysis

Summary statistics from each phenotype were analyzed by MAGENTA (44) to identify any enriched association in gene pathways for canonical gene sets and Gene Ontology gene sets (25). The original databases used were acquired from the Molecular Signatures Database (version MSigDB v6.1, 45) and were then modified for compatibility. An enrichment cut-off for the 75th percentile was applied as recommended for highly polygenic traits (44). A 5% FDR was applied to results to correct for multiple testing.

### Gene-based association

Summary statistics from our GWAS were used as input for analysis by S-PrediXcan (29) to test for association between phenotype and whole gene expression. As with the eQTL analysis, results were limited to fibroblast tissue, as it is a functionally relevant model due to the high density of keratocytes, a sub-type of fibroblast, within the cornea (26). A Bonferroni corrected significance threshold of *P* < 2.5 × 10^−7^ is recommended when testing all gene-tissue pairs. However, as we only included fibroblast tissue in this analysis, this value was adjusted to correct for all tested gene-tissue pairs and set at *P* < 5.5 × 10^−6^.

### Replication of associated markers

Autosomal SNPs with independent association in this study, as identified by conditional analysis, were subject to replication testing with Bonferroni correction for multiple testing. The replication was conducted with a meta-analysis in two independent cohorts, EPIC-Norfolk (46,47) and TwinsUK (48). In brief, GWAS and subsequent meta-analysis was conducted for both CH and CRF separately in these cohorts. In total 9029 participants of European ancestry were included in these analyses, with no known sample overlap with the UK Biobank. Full details of this analysis are described elsewhere (7).

### Mendelian randomization

Mendelian randomization was used to test for causal inference between IOPcc with CH and CRF. Summary SNP data for IOPcc were used from Khawaja *et al*.’s (18) recent meta-analysis (which included the UK Biobank). The lead SNPs from each separate genomic region were selected as genetic instruments for the exposure variable in order to prevent confounding errors resulting from correlated SNPs. Any SNPs significantly associated with the outcome variable (*P* < 5 × 10^−8^) were excluded as they may have a common cause with the outcome (the MR independence assumption). Standard regression, penalized regression, robust regression, and penalized robust regression models were applied for these analyses with results indicating that the penalized models were the most suitable. The penalized models downweigh the contribution of variants with heterogenous causal estimates to the analysis; due to the inclusion of a relatively large number of instrument variables in these analyses, a penalized regression is more stable than a standard linear regression (49). For the inverse-variance models, the psi variable was set as the observational correlation between the exposure and outcome phenotypes to account for sample overlap. All analysis was performed in the MendelianRandomization (50) package in R (40).

## Funding

The TwinsUK study was funded by the Wellcome Trust (105022/Z/14/Z). The study also receives support from the National Institute for Health Research (NIHR)-funded BioResource, Clinical Research Facility and Biomedical Research Centre based at Guy’s and St Thomas’ NHS Foundation Trust in partnership with King’s College London. SNP genotyping was performed by the Wellcome Trust Sanger Institute and National Eye Institute via NIH/CIDR. M.S. is supported by the Wellcome Trust (Grant 206619/Z/17/Z).

The EPIC-Norfolk study (https://doi.org/10.22025/2019.10.105. 00004) has received funding from the Medical Research Council (MR/N003284/1 and MC-UU_12015/1) and Cancer Research UK (C864/A14136). The genetics work in the EPIC-Norfolk study was funded by the Medical Research Council (MC_PC_13048).We are grateful to all the participants who have been part of the project and to the many members of the study teams at the University of Cambridge who have enabled this research. A.P.K. is supported by a Moorfields Eye Charity Career Development Fellowship.

## Supplementary Material

Supplementary_Figure_1a_ddaa155

Supplementary_Figure_1b_ddaa155

Suplementary_list_ddaa155

Supplementary_data_ddaa155

## References

[1] Luce D.A. (2005) Determining in vivo biomechanical properties of the cornea with an ocular response analyzer. J Cataract Refract Surg, 31, 156–162. 15721708 10.1016/j.jcrs.2004.10.044

[2] Garcia-Porta N., Fernandes P., Queiros A., Salgado-Borges J., Parafita-Mato M. and Gonzalez-Meijome J.M. (2014) Corneal biomechanical properties in different ocular conditions and new measurement techniques. ISRN Ophthalmol., 2014, 724546. 24729900 10.1155/2014/724546 PMC3960740

[3] Luce D. (2006) Methodology for cornea compensated IOP and corneal resistance factor for the Reichert ocular response Analyzer. Invest. Ophthalmol. Vis. Sci., 47, 2266–2266.

[4] Medeiros F.A., Meira-Freitas D., Lisboa R., Kuang T.M., Zangwill L.M. and Weinreb R.N. (2013) Corneal hysteresis as a risk factor for glaucoma progression: a prospective longitudinal study. Ophthalmology, 120, 1533–1540. 23642371 10.1016/j.ophtha.2013.01.032 PMC3804228

[5] Medeiros F.A. and Weinreb R.N. (2006) Evaluation of the influence of corneal biomechanical properties on intraocular pressure measurements using the ocular response analyzer. J. Glaucoma, 15, 364–370. 16988597 10.1097/01.ijg.0000212268.42606.97

[6] Liu J. and Roberts C.J. (2005) Influence of corneal biomechanical properties on intraocular pressure measurement: quantitative analysis. J Cataract Refract Surg, 31, 146–155. 15721707 10.1016/j.jcrs.2004.09.031

[7] Khawaja A.P., Rojas Lopez K.E., Hardcastle A.J., Hammond C.J., Liskova P., Davidson A.E., Gore D.M., Hafford Tear N.J., Pontikos N., Hayat S. et al. (2019) Genetic variants associated with corneal biomechanical properties and potentially conferring susceptibility to keratoconus in a genome-wide association study. JAMA Ophthalmol., 137, 1005–1012. 10.1001/jamaophthalmol.2019.2058 PMC6604088 31246245

[8] del Buey M.A., Cristobal J.A., Ascaso F.J., Lavilla L. and Lanchares E. (2009) Biomechanical properties of the cornea in Fuchs' corneal dystrophy. Invest. Ophthalmol. Vis. Sci., 50, 3199–3202. 19255149 10.1167/iovs.08-3312

[9] Carbonaro F., Andrew T., Mackey D.A., Spector T.D. and Hammond C.J. (2008) The heritability of corneal hysteresis and ocular pulse amplitude: a twin study. Ophthalmology, 115, 1545–1549. 18439682 10.1016/j.ophtha.2008.02.011

[10] Allen N.E., Sudlow C., Peakman T. and Collins R. (2014) UK biobank data: come and get it. Sci. Transl. Med., 6, 224ed4. 10.1126/scitranslmed.3008601 24553384

[11] Zhang B., Shweikh Y., Khawaja A.P., Gallacher J., Bauermeister S., Foster P.J., Eye U.K. and Vision C. (2019) Associations with corneal hysteresis in a population cohort: results from 96 010 UK biobank participants. Ophthalmology, 126, 1500–1510. 31471087 10.1016/j.ophtha.2019.06.029

[12] Bolivar G., Sanchez-Barahona C., Teus M., Castejon M.A., Paz-Moreno-Arrones J., Gutierrez-Ortiz C. and Mikropoulos D.G. (2015) Effect of topical prostaglandin analogues on corneal hysteresis. Acta Ophthalmol., 93, e495–e498. 25722009 10.1111/aos.12689

[13] Devlin B. and Roeder K. (1999) Genomic control for association studies. Biometrics, 55, 997–1004. 11315092 10.1111/j.0006-341x.1999.00997.x

[14] Bulik-Sullivan B.K., Loh P.R., Finucane H.K., Ripke S., Yang J., Schizophrenia Working Group of the Psychiatric Genomics, C, Patterson N., Daly M.J., Price A.L. and Neale B.M. (2015) LD score regression distinguishes confounding from polygenicity in genome-wide association studies. Nat. Genet., 47, 291–295. 25642630 10.1038/ng.3211 PMC4495769

[15] Zimmermann D.R., Trueb B., Winterhalter K.H., Witmer R. and Fischer R.W. (1986) Type-vi collagen is a major component of the human cornea. FEBS Lett., 197, 55–58. 3512309 10.1016/0014-5793(86)80297-6

[16] Muragaki Y., Mattei M.G., Yamaguchi N., Olsen B.R. and Ninomiya Y. (1991) The complete primary structure of the human alpha-1(Viii) chain and assignment of its gene (Col8a1) to chromosome-3. Eur. J. Biochem., 197, 615–622. 2029894 10.1111/j.1432-1033.1991.tb15951.x

[17] West-Mays J.A. and Dwivedi D.J. (2006) The keratocyte: corneal stromal cell with variable repair phenotypes. Int. J. Biochem. Cell B, 38, 1625–1631. 10.1016/j.biocel.2006.03.010 PMC2505273 16675284

[18] Khawaja A.P., Cooke Bailey J.N., Wareham N.J., Scott R.A., Simcoe M., Igo R.P. Jr., Song Y.E., Wojciechowski R., Cheng C.Y., Khaw P.T. et al. (2018) Genome-wide analyses identify 68 new loci associated with intraocular pressure and improve risk prediction for primary open-angle glaucoma. Nat. Genet., 50, 778–782. 29785010 10.1038/s41588-018-0126-8 PMC5985943

[19] Brezin A.P., Nedelec B., Barjol A., Rothschild P.R., Delpech M. and Valleix S. (2011) A new VCAN/versican splice acceptor site mutation in a French Wagner family associated with vascular and inflammatory ocular features. Mol. Vis., 17, 1669–1678. 21738396 PMC3130719

[20] Amberger J., Bocchini C. and Hamosh A. (2011) A new face and new challenges for online mendelian inheritance in man (OMIM (R)). Hum. Mutat., 32, 564–567. 21472891 10.1002/humu.21466

[21] Rong S.S., Ma S.T.U., Yu X.T., Ma L., Chu W.K., Chan T.C.Y., Wang Y.M., Young A.L., Pang C.P., Jhanji V. et al. (2017) Genetic associations for keratoconus: a systematic review and meta-analysis. Sci. Rep., 7, 4620. 28676647 10.1038/s41598-017-04393-2 PMC5496893

[22] Afshari N.A., Igo R.P., Morris N.J., Stambolian D., Sharma S., Pulagam V.L., Dunn S., Stamler J.F., Truitt B.J., Rimmler J. et al. (2017) Genome-wide association study identifies three novel loci in Fuchs endothelial corneal dystrophy. Nat. Commun., 8, 14898. 10.1038/ncomms14898 PMC5379100 28358029

[23] McLaren W., Gil L., Hunt S.E., Riat H.S., Ritchie G.R., Thormann A., Flicek P. and Cunningham F. (2016) The ensembl variant effect predictor. Genome Biol., 17, 122. 27268795 10.1186/s13059-016-0974-4 PMC4893825

[24] Naba A., Clauser K.R., Hoersch S., Liu H., Carr S.A. and Hynes R.O. (2012) The matrisome: in silico definition and in vivo characterization by proteomics of normal and tumor extracellular matrices. Mol. Cell. Proteomics, 11, M111 014647. 10.1074/mcp.M111.014647 PMC3322572 22159717

[25] Ashburner M., Ball C.A., Blake J.A., Botstein D., Butler H., Cherry J.M., Davis A.P., Dolinski K., Dwight S.S., Eppig J.T. et al. (2000) Gene ontology: tool for the unification of biology. Nat. Genet., 25, 25–29. 10802651 10.1038/75556 PMC3037419

[26] Patel S.V., McLaren J.W., Hodge D.O. and Bourne W.M. (2001) Normal human keratocyte density and corneal thickness measurement by using confocal microscopy in vivo. Invest. Ophthalmol. Vis. Sci., 42, 333–339. 11157863

[27] Consortium G.T. (2015) Human genomics. The genotype-tissue expression (GTEx) pilot analysis: multitissue gene regulation in humans. Science, 348, 648–660. 25954001 10.1126/science.1262110 PMC4547484

[28] Carnes M.U., Allingham R.R., Ashley-Koch A. and Hauser M.A. (2018) Transcriptome analysis of adult and fetal trabecular meshwork, cornea, and ciliary body tissues by RNA sequencing. Exp. Eye Res., 167, 91–99. 27914989 10.1016/j.exer.2016.11.021

[29] Barbeira A.N., Dickinson S.P., Bonazzola R., Zheng J., Wheeler H.E., Torres J.M., Torstenson E.S., Shah K.P., Garcia T., Edwards T.L. et al. (2018) Exploring the phenotypic consequences of tissue specific gene expression variation inferred from GWAS summary statistics. Nat. Commun., 9, 1825. 29739930 10.1038/s41467-018-03621-1 PMC5940825

[30] Smith G.D. and Ebrahim S. (2003) Mendelian randomization': can genetic epidemiology contribute to understanding environmental determinants of disease? Int. J. Epidemiol., 32, 1–22. 12689998 10.1093/ije/dyg070

[31] Wise A.L., Gyi L. and Manolio T.A. (2013) eXclusion: toward integrating the X chromosome in genome-wide association analyses. Am. J. Hum. Genet., 92, 643–647. 23643377 10.1016/j.ajhg.2013.03.017 PMC3644627

[32] Iacob S. and Cs-Szabo G. (2010) Biglycan regulates the expression of EGF receptors through EGF signaling pathways in human articular chondrocytes. Connect. Tissue Res., 51, 347–358. 20367117 10.3109/03008200903427695

[33] Shah S., Laiquzzaman M., Bhojwani R., Mantry S. and Cunliffe I. (2007) Assessment of the biomechanical properties of the cornea with the ocular response analyzer in normal and keratoconic eyes. Invest. Ophthalmol. Vis. Sci., 48, 3026–3031. 17591868 10.1167/iovs.04-0694

[34] Luz A., Fontes B., Ramos I.C., Lopes B., Correia F., Schor P. and Ambrósio R. (2012) Evaluation of ocular biomechanical indices to distinguish normal from keratoconus eyes. Int. J. Kerat. Ect. Cor. Dis., 1, 145–150.

[35] Gilchrist J.M. (1996) On the precision and reliability of IOP measurements. Br. J. Ophthalmol., 80, 586–587. 8795367 10.1136/bjo.80.7.586 PMC505549

[36] McCarthy S., Das S., Kretzschmar W., Delaneau O., Wood A.R., Teumer A., Kang H.M., Fuchsberger C., Danecek P., Sharp K. et al. (2016) A reference panel of 64,976 haplotypes for genotype imputation. Nat. Genet., 48, 1279–1283. 27548312 10.1038/ng.3643 PMC5388176

[37] Huang J., Howie B., McCarthy S., Memari Y., Walter K., Min J.L., Danecek P., Malerba G., Trabetti E., Zheng H.F. et al. (2015) Improved imputation of low-frequency and rare variants using the UK10K haplotype reference panel. Nat. Commun., 6, 8111. 10.1038/ncomms9111 PMC4579394 26368830

[38] Bycroft C., Freeman C., Petkova D., Band G., Elliott L.T., Sharp K., Motyer A., Vukcevic D., Delaneau O., O'Connell J. et al. (2018) The UK biobank resource with deep phenotyping and genomic data. Nature, 562, 203–209. 30305743 10.1038/s41586-018-0579-z PMC6786975

[39] Loh P.R., Tucker G., Bulik-Sullivan B.K., Vilhjalmsson B.J., Finucane H.K., Salem R.M., Chasman D.I., Ridker P.M., Neale B.M., Berger B. et al. (2015) Efficient Bayesian mixed-model analysis increases association power in large cohorts. Nat. Genet., 47, 284. 25642633 10.1038/ng.3190 PMC4342297

[40] R Core Team (2017) R Foundation for Statistical Computing, Vol. 3.4.0, Vienna, Austria.

[41] Zheng J., Erzurumluoglu A.M., Elsworth B.L., Kemp J.P., Howe L., Haycock P.C., Hemani G., Tansey K., Laurin C., Early G. et al. (2017) LD hub: a centralized database and web interface to perform LD score regression that maximizes the potential of summary level GWAS data for SNP heritability and genetic correlation analysis. Bioinformatics, 33, 272–279. 27663502 10.1093/bioinformatics/btw613 PMC5542030

[42] Bulik-Sullivan B., Finucane H.K., Anttila V., Gusev A., Day F.R., Loh P.R., Duncan L., Perry J.R.B., Patterson N., Robinson E.B. et al. (2015) An atlas of genetic correlations across human diseases and traits. Nat. Genet., 47, 1236. 26414676 10.1038/ng.3406 PMC4797329

[43] Yang J., Lee S.H., Goddard M.E. and Visscher P.M. (2011) GCTA: a tool for genome-wide complex trait analysis. Am. J. Hum. Genet., 88, 76–82. 21167468 10.1016/j.ajhg.2010.11.011 PMC3014363

[44] Segre A.V., Consortium, D., Investigators, M., Groop, L., Mootha V.K., Daly M.J. and Altshuler D. (2010) Common inherited variation in mitochondrial genes is not enriched for associations with type 2 diabetes or related glycemic traits. PLoS Genet., 6, e1001058. 20714348 10.1371/journal.pgen.1001058 PMC2920848

[45] Liberzon A., Subramanian A., Pinchback R., Thorvaldsdottir H., Tamayo P. and Mesirov J.P. (2011) Molecular signatures database (MSigDB) 3.0. Bioinformatics, 27, 1739–1740. 21546393 10.1093/bioinformatics/btr260 PMC3106198

[46] Hayat S.A., Luben R., Keevil V.L., Moore S., Dalzell N., Bhaniani A., Khawaja A.P., Foster P., Brayne C., Wareham N.J. et al. (2014) Cohort profile: a prospective cohort study of objective physical and cognitive capability and visual health in an ageing population of men and women in Norfolk (EPIC-Norfolk 3). Int. J. Epidemiol., 43, 1063–1072. 23771720 10.1093/ije/dyt086 PMC4121549

[47] Khawaja A.P., Chan M.P., Hayat S., Broadway D.C., Luben R., Garway-Heath D.F., Sherwin J.C., Yip J.L., Dalzell N., Wareham N.J. et al. (2013) The EPIC-Norfolk Eye study: rationale, methods and a cross-sectional analysis of visual impairment in a population-based cohort. BMJ Open, 3, e002684. 10.1136/bmjopen-2013-002684 PMC3612817 23516272

[48] Moayyeri A., Hammond C.J., Valdes A.M. and Spector T.D. (2013) Cohort profile: TwinsUK and healthy ageing twin study. Int. J. Epidemiol., 42, 76–85. 22253318 10.1093/ije/dyr207 PMC3600616

[49] Bowden J., Davey Smith G., Haycock P.C. and Burgess S. (2016) Consistent estimation in mendelian randomization with some invalid instruments using a weighted median estimator. Genet. Epidemiol., 40, 304–314. 27061298 10.1002/gepi.21965 PMC4849733

[50] Burgess S. and Thompson S.G. (2017) Interpreting findings from mendelian randomization using the MR-egger method. Eur. J. Epidemiol., 32, 377–389. 28527048 10.1007/s10654-017-0255-x PMC5506233

