# Supplementary figures and images for: Genome-wide association study of corneal biomechanical properties identifies over 200 loci providing insight into the genetic etiology of ocular diseases
Source: Hum Mol Genet. 2020 Jul 27;29(18):3154–64. doi: 10.1093/hmg/ddaa155 (PMC7645703; doi:10.1093/hmg/ddaa155)

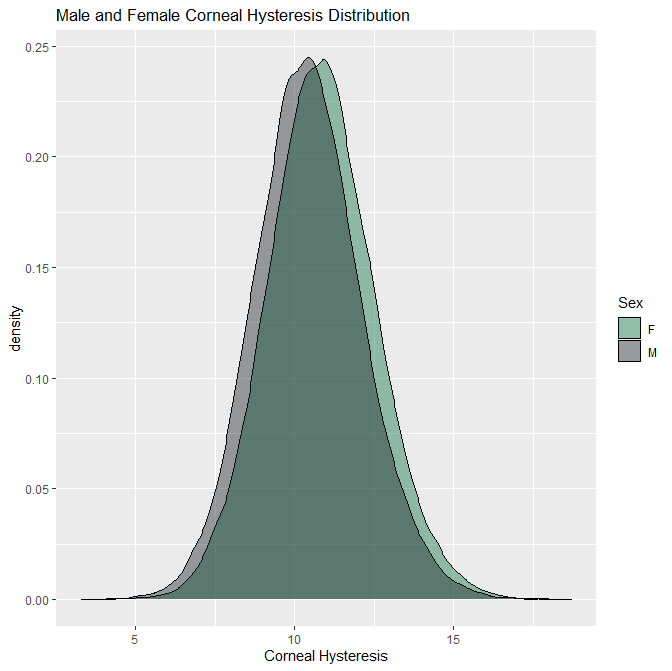

Supplement: Supplementary_Figure_1a_ddaa155 [file supplementary_figure_1a_ddaa155.png]

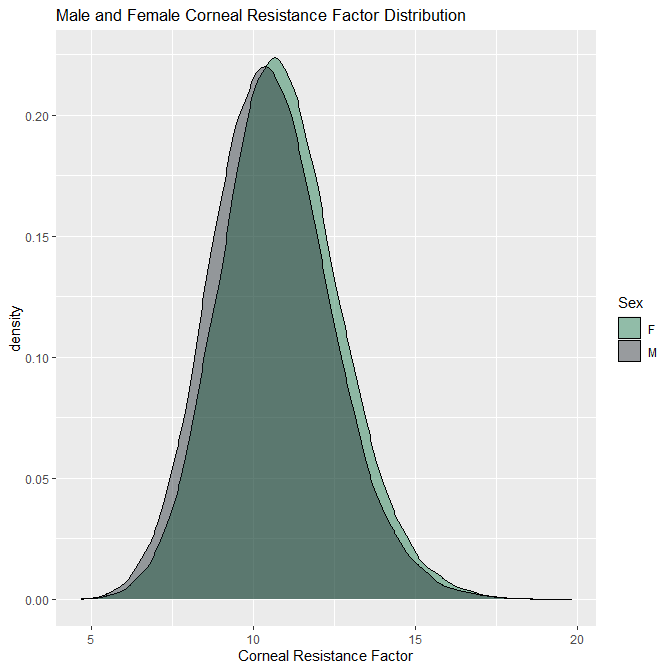

Supplement: Supplementary_Figure_1b_ddaa155 [file supplementary_figure_1b_ddaa155.png]
