# Supplementary material for: Genome-wide association study of corneal biomechanical properties identifies over 200 loci providing insight into the genetic etiology of ocular diseases
Source: Hum Mol Genet. 2020 Jul 27;29(18):3154–64. doi: 10.1093/hmg/ddaa155 (PMC7645703; doi:10.1093/hmg/ddaa155)
Supplement: Suplementary_list_ddaa155 [file suplementary_list_ddaa155.docx]

**Members of the UK Biobank Eye and Vision Consortium Membership**

• Prof Tariq ASLAM - Manchester University, Manchester, United Kingdom

• Prof Sarah BARMAN - Kingston University, London, United Kingdom

• Prof Jenny BARRETT - University of Leeds, Yorkshire, United Kingdom

• Prof Paul BISHOP - Manchester University, Manchester, United Kingdom

• Mr Peter BLOWS - NIHR Biomedical Research Centre, Moorfields Eye Hospital NHS Foundation Trust and UCL Institute of Ophthalmology, London, United Kingdom

• Dr Catey BUNCE - King’s College London, London, United Kingdom

• Dr Roxana CARARE - University of Southampton, Southampton, United Kingdom

• Prof Usha CHAKRAVARTHY - Queens University Belfast, Belfast, Ireland

• Miss Michelle CHAN - NIHR Biomedical Research Centre, Moorfields Eye Hospital NHS Foundation Trust and UCL Institute of Ophthalmology, London, United Kingdom

• Dr Sharon CHUA - NIHR Biomedical Research Centre, Moorfields Eye Hospital NHS Foundation Trust and UCL Institute of Ophthalmology, London, United Kingdom

• Prof David CRABB – City, University of London, London, United Kingdom

• Mrs Philippa CUMBERLAND - UCL Great Ormond Street Institute of Child Health, London, United Kingdom

• Dr Alexander DAY - NIHR Biomedical Research Centre, Moorfields Eye Hospital NHS Foundation Trust and UCL Institute of Ophthalmology, London, United Kingdom

• Miss Parul DESAI - NIHR Biomedical Research Centre, Moorfields Eye Hospital NHS Foundation Trust and UCL Institute of Ophthalmology, London, United Kingdom

• Prof Bal DHILLON - University of Edinburgh, Scotland, United Kingdom

• Prof Andrew DICK - University of Bristol, Bristol, United Kingdom

• Dr Cathy EGAN - NIHR Biomedical Research Centre, Moorfields Eye Hospital NHS Foundation Trust and UCL Institute of Ophthalmology, London, United Kingdom

• Prof Sarah ENNIS - University of Southampton, Southampton, United Kingdom

• Prof Paul FOSTER - NIHR Biomedical Research Centre, Moorfields Eye Hospital NHS Foundation Trust and UCL Institute of Ophthalmology, London, United Kingdom

• Dr Marcus FRUTTIGER - NIHR Biomedical Research Centre, Moorfields Eye Hospital NHS Foundation Trust and UCL Institute of Ophthalmology, London, United Kingdom

• Dr John GALLACHER - University of Oxford, Oxford, United Kingdom

• Prof David (Ted) GARWAY-HEATH - NIHR Biomedical Research Centre, Moorfields Eye Hospital NHS Foundation Trust and UCL Institute of Ophthalmology, London, United Kingdom

• Dr Jane GIBSON - University of Southampton, Southampton, United Kingdom

• Mr Dan GORE - NIHR Biomedical Research Centre, Moorfields Eye Hospital NHS Foundation Trust and UCL Institute of Ophthalmology, London, United Kingdom

• Prof Jeremy GUGGENHEIM - Cardiff University, Wales, United Kingdom

• Prof Chris HAMMOND - King's College London, London, United Kingdom

• Prof Alison HARDCASTLE - NIHR Biomedical Research Centre, Moorfields Eye Hospital NHS Foundation Trust and UCL Institute of Ophthalmology, London, United Kingdom

• Prof Simon HARDING - University of Liverpool, London, United Kingdom

• Dr Ruth HOGG - Queens University Belfast, Belfast, Ireland

• Dr Pirro HYSI - King's College London, London, United Kingdom

• Mr Pearse A KEANE - NIHR Biomedical Research Centre, Moorfields Eye Hospital NHS Foundation Trust and UCL Institute of Ophthalmology, London, United Kingdom

• Prof Sir Peng Tee KHAW - NIHR Biomedical Research Centre, Moorfields Eye Hospital NHS Foundation Trust and UCL Institute of Ophthalmology, London, United Kingdom

• Mr Anthony KHAWAJA - NIHR Biomedical Research Centre, Moorfields Eye Hospital NHS Foundation Trust and UCL Institute of Ophthalmology, London, United Kingdom

• Mr Gerassimos LASCARATOS - NIHR Biomedical Research Centre, Moorfields Eye Hospital NHS Foundation Trust and UCL Institute of Ophthalmology, London, United Kingdom

• Prof Andrew LOTERY- University of Southampton, Southampton, United Kingdom

• Prof Phil LUTHERT - NIHR Biomedical Research Centre, Moorfields Eye Hospital NHS Foundation Trust and UCL Institute of Ophthalmology, London, United Kingdom

• Dr Tom MACGILLIVRAY - University of Edinburgh, Scotland, United Kingdom

• Dr Sarah MACKIE - University of Leeds, Yorkshire, United Kingdom

• Prof Keith MARTIN - University of Cambridge, Cambridge, United Kingdom

• Ms Michelle MCGAUGHEY - Queen’s University Belfast, Belfast, Ireland

• Dr Bernadette MCGUINNESS - Queen’s University Belfast, Belfast, Ireland

• Dr Gareth MCKAY - Queen's University Belfast, Belfast, Ireland

• Mr Martin MCKIBBIN - Leeds Teaching Hospitals NHS Trust, Yorkshire, United Kingdom

• Dr Danny MITRY – NIHR Biomedical Research Centre, Moorfields Eye Hospital NHS Foundation Trust and UCL Institute of Ophthalmology, London, United Kingdom & Royal Free Hospital, London, United Kingdom

• Prof Tony MOORE - NIHR Biomedical Research Centre, Moorfields Eye Hospital NHS Foundation Trust and UCL Institute of Ophthalmology, London, United Kingdom

• Prof James MORGAN - Cardiff University, Wales, United Kingdom

• Ms Zaynah MUTHY – NIHR Biomedical Research Centre, Moorfields Eye Hospital NHS Foundation Trust and UCL Institute of Ophthalmology, London, United Kingdom

• Mr Eoin O'SULLIVAN - King's College Hospital NHS Foundation Trust, London, United Kingdom

• Dr Chris OWEN - St George's, University of London, London, United Kingdom

• Mr Praveen PATEL - NIHR Biomedical Research Centre, Moorfields Eye Hospital NHS Foundation Trust and UCL Institute of Ophthalmology, London, United Kingdom

• Mr Euan PATERSON - Queens University Belfast, Belfast, Ireland

• Dr Tunde PETO - Queen's University Belfast, Belfast, Ireland

• Dr Axel PETZOLD - UCL Institute of Neurology, London, United Kingdom

• Prof Jugnoo RAHI - UCL Great Ormond Street Institute of Child Health, London, United Kingdom

• Dr Alicja RUDNICKA - St George's, University of London, London, United Kingdom

• Mr Jay SELF - University of Southampton, Southampton, United Kingdom

• Prof Sobha SIVAPRASAD - NIHR Biomedical Research Centre, Moorfields Eye Hospital NHS Foundation Trust and UCL Institute of Ophthalmology, London, United Kingdom

• Mr David STEEL - Newcastle University, Newcastle, United Kingdom

• Mrs Irene STRATTON - Gloucestershire Hospitals NHS Foundation Trust

• Mr Nicholas STROUTHIDIS - NIHR Biomedical Research Centre, Moorfields Eye Hospital NHS Foundation Trust and UCL Institute of Ophthalmology, London, United Kingdom

• Prof Cathie SUDLOW - University of Edinburgh, Scotland, United Kingdom

• Dr Caroline THAUNG - NIHR Biomedical Research Centre, Moorfields Eye Hospital NHS Foundation Trust and UCL Institute of Ophthalmology, London, United Kingdom

• Miss Dhanes THOMAS - NIHR Biomedical Research Centre, Moorfields Eye Hospital NHS Foundation Trust and UCL Institute of Ophthalmology, London, United Kingdom

• Prof Emanuele TRUCCO - University of Dundee, Scotland, United Kingdom

• Prof Adnan TUFAIL - NIHR Biomedical Research Centre, Moorfields Eye Hospital NHS Foundation Trust and UCL Institute of Ophthalmology, London, United Kingdom

• Dr Veronique VITART - University of Edinburgh, Scotland, United Kingdom

• Prof Stephen VERNON – Nottingham University Hospitals NHS Trust, Nottingham, United Kingdom

• Mr Ananth VISWANATHAN - NIHR Biomedical Research Centre, Moorfields Eye Hospital NHS Foundation Trust and UCL Institute of Ophthalmology, London, United Kingdom

• Miss Cathy WILLIAMS - University of Bristol, Bristol, United Kingdom

• Dr Katie WILLIAMS - King's College London, London, United Kingdom

• Prof Jayne WOODSIDE - Queen's University Belfast, Belfast, Ireland

• Dr Max YATES - University of East Anglia, Norwich, United Kingdom

• Ms Jennifer YIP - University of Cambridge, Cambridge, United Kingdom

• Dr Yalin ZHENG - University of Liverpool, London, United Kingdom
